# Systems Biology in Aging: Linking the Old and the Young

**DOI:** 10.2174/138920212803251418

**Published:** 2012-11

**Authors:** Lei Hou, Jialiang Huang, Christopher D Green, Jerome Boyd-Kirkup, Wei Zhang, Xiaoming Yu, Wenxuan Gong, Bing Zhou, Jing-Dong J Han

**Affiliations:** 1 Chinese Academy of Sciences Key Laboratory of Computational Biology, Chinese Academy of Sciences-Max Planck Partner Institute for Computational Biology, Shanghai Institutes for Biological Sciences, Chinese Academy of Sciences, Shanghai, China; 2 Center of Molecular Systems Biology, Institute of Genetics and Developmental Biology, Chinese Academy of Sciences, Beijing, China

**Keywords:** Systems biology, Genomics, Proteomics, Longevity, Aging, Network analysis.

## Abstract

Aging can be defined as a process of progressive decline in the physiological capacity of an organism, manifested by accumulated alteration and destabilization at the whole system level. Systems biology approaches offer a promising new perspective to examine the old problem of aging. We begin this review by introducing the concepts of systems biology, and then illustrate the application of systems biology approaches to aging research, from gene expression profiling to network analysis. We then introduce the network that can be constructed using known lifespan and aging regulators, and conclude with a look forward to the future of systems biology in aging research. In summary, systems biology is not only a young field that may help us understand aging at a higher level, but also an important platform that can link different levels of knowledge on aging, moving us closer to a more comprehensive control of systematic decline during aging.

## 1 INTRODUCTION

Aging has fascinated researchers since ancient times. The hugely complicated process that has been revealed may be interpreted from different aspects, such as the accumulation of oxidative damage, shortening of telomeres, the costs of reproduction, metabolic rates, cellular senescence, etc., and these have in turn given rise to diverse theories of aging [1]. However, thanks to forward and reverse genetic technologies, researchers in the recent decades have established that despite its complexity, a single or a few key genes in a few key pathways can modulate the aging rate. The most important players would appear to be those in nutrient sensing pathways or stress response pathways, such as DAF-2/IGF1R and DAF-16/FOXO in the Insulin/IGF like signaling pathway, AAK-2/AMPK in another nutrient sensing pathway, JNK in the stress response pathway, LET-363/mTOR as an inhibitor of autophagy and activator of translation and SIRT1/SIR2 in genome stability maintenance, to name a few [2, 3]. In addition to genetic perturbations, dietary perturbations, such as diet restriction (DR) are known to significantly extend lifespan in most organisms examined from yeasts to primates, although different pathways may act under different DR conditions, and alternative DR strategies also effect *C.elegans* lifespan in different ways [3, 4]. The main pathways revealed under different DR regimens are summarized in Fig. (**1**). In this small, convoluted DR response network, DAF-16 and ceTOR/LET-363 seem to play a central role by integrating upstream signals and regulating downstream processes. The crosstalk between these pathways and the combination of regulatory signals ultimately affect the systems level phenotype of aging and aging-associated functional decline. From this aspect, systems biology may be critical to integrate the various aging regulatory signals, and predict and interpret the systems level output, i.e. the aging-associated phenotypes.

Systems biology examines a biological individual as a living system, consisting of components of different layers Fig. (**2A**): 1) the chromosomal level including genomic sequences and the epigenetic states of chromosomes, such as DNA methylation and histone modifications, 2) the RNA level including mRNA and non-coding RNAs, 3) the protein level including proteins and their different modification states, and 4) the metabolite level. The cellular states at these different layers can be monitored by high-throughput experimental approaches such as next generation sequencing and de novo assembly for genomic sequences, chromatin immunoprecipitation followed by microarray (ChIP-chip) or deep sequencing (ChIP-seq) for histone modifications, bisulfite sequencing for DNA methylation, microarray or RNAseq for mRNA and for miRNA, protein microarray or mass spectrometry (MS) for proteins, and MS or nuclear magnetic resonance (NMR) for metabolites. Molecular interactions within and between these layers give rise to the observed states of the whole system, manifested as phenotypes Fig. (**2**). All of these layers, as well as other potentially unidentified ones, can be perturbed or regulated by physiological signals, as well as environmental cues. Unlike classical approaches, which focus on only specific molecules or pathways, systems biology takes advantage of these highthroughput approaches to study components in each layer and their interactions at multiple layers during a time course or after perturbation.

## 2 DETECTING MOLECULAR AGING PROFILES USING HIGH-THROUGHPUT APPROACHES

To examine the changes of individual genes during aging, experiments have been designed either to compare young and old samples, or to monitor a time series during aging. In some cases, different conditions (different genetic backgrounds, tissues, diets, stimuli, etc.) are also taken into consideration. These data are then analyzed to identify differentially expressed genes, age-related gene expression changes, and functional enrichment among the changed genes.

As early as 1999, Lee *et al.* [7] showed an increase of expression in stress response genes and a decrease in metabolic and biosynthetic genes during aging. Samples were collected from skeletal muscle of mice to compare old and young animals under normal and DR conditions [7]. They found that DR could postpone aging by inhibiting macromolecular damage and promoting protein turnover. Pioneering microarray studies prior to 2004,suggested that the effects of aging, DR and exercise are characterized by transcriptional changes [8]. Lund *et al.,* [9] using worms from different genetic backgrounds, found that the expression of heat shock genes decreased while certain transposases increased during *C.* *elegans* aging. Their work also suggested that the aging process and the transition from the reproductive to the dauer stage share some similar alterations in gene expression. Pletcher *et al.* [10] showed that genes related to stress response and oogenesis seem to change during *Drosophila melanogaster* aging, and those involved in cell growth, metabolism and reproduction are down-regulated by DR. This data argued against the hypothesis that aging is due to transcriptional changes in some specific genomic regions, but rather support it as the result of increasing disregulation of gene expression. Kayo *et al.* [11] using samples from the muscles of monkeys, found an increase in inflammation and oxidative stress as well as a decrease in mitochondrial electron transport and oxidative phosphorylation during aging. Bronikowski *et al.* [12] demonstrated that, in mouse heart, exercise could delay the aging-related expression changes, which involve inflammatory response, stress response, signal transduction and energy metabolism genes. Studies during this period also provided the first glimpse of aging-related global transcriptomic changes. They indicated that some common changes, such as the involvement of stress response, energy metabolism and mitochondrial genes, are shared by different species during aging and modified by DR.

Since 2004, many more factors have been considered when designing microarray experiments. For example, to compare age-related changes between different species, McCarroll *et al.* [13] carried out microarray analyses for both *C.elegans* and *D. melanogaster*, and by examining changes in orthologous genes between the two species they detected similar age-related changes in mitochondria and DNA repair genes in both organisms. To examine tissue specific changes, Zahn *et al.* [14] generated expression profiles for 16 different mouse tissues during aging, which are collectively deposited in the AGEMAP database, and identified some genes that have different age-related patterns in different groups of tissues. Rather than focus on spatial differences, others have investigated temporal differences in more detail with additional time points, and have extended the studies into humans. Lu *et al.* [15] provided the first comprehensive aging-related map of the human brain transcriptome changes using 30 postmortem samples from age 26 to 106, and identified that, among many other molecular and functional changes, DNA repair related genes significantly increase with age, suggesting that there is DNA damage stress during human brain aging. Also, using time course data analyses, Somel *et al.* [16, 17] found a close relationship between development and aging, and a delay in the timing of the aging transition in humans compared with other primates. Recently using 1340 tissue samples from 57 developing and adult brain samples, Kang *et al*. [18] quantified expression trajectories at both the gene-level and exon-level, and provide a rich resource on both temporal and spatial changes in human brain. 

In order to find common pathways affected by different dietary interventions that modulate aging and lifespan, we have obtained midlife hepatic gene expression profiles of mice on high-fat diet, high-fat diet with CR, high-fat diet with voluntary exercise, low-fat diet, low-fat diet with CR and low-fat diet with voluntary exercises. We found that pathways whose gene expression levels are correlated with the mean lifespan under these six conditions are enriched for aging regulatory pathways, suggesting different dietary intervention regimens may target a set of common lifespan modulating pathways and functions [19]. Similar to gene expression profiles for coding genes, aging-related changes in miRNAs have also been profiled for *C. elegans *[20, 21], monkey and the human brain [17, 20, 21]. 

Other high-throughput data has helped to globally characterize aging from different perspectives. As protein translation plays a regulatory role in aging and lifespan, recent deep sequence-based polysome-RNA profiling has been used to detect translationally regulated genes through eukaryotic translation initiation factor 4G (eIF4G) knock-down, known to extend lifespan, and has revealed that many lifespan regulators, in particular genes encoding respiratory chain components are controlled at the translation level [22, 23]. 

These high throughput profiling experiments have generated large amounts of data for meta-analysis [24], which can compare molecular functions and expression patterns that change during aging in different systems. However, such studies are far from exhaustive, as they only describe the molecular changes during aging, which could in fact be the consequence of aging, rather than the cause of aging. Thus to explore the causal factors for aging, studies are increasingly devoted to the identification of aging and lifespan regulators.

## 3 INFERRING AGING REGULATORS

The majority of the known aging/lifespan regulators, for example, *age-1* [25], *daf-2* and *daf-16* [26], were identified through genetics approaches in model organisms such as yeast, worm and fruitfly. Most of these genes are so defined because their perturbation (knock out, knock down or over-expression) can extend lifespan. A few genome-wide RNAi screens have also identified such genes (see the accompanying review by Bennett *et al*. [27] in this issue). Searching for aging regulators is important to reduce the complexity of aging down to single genes. This is crucial to delineating how aging is regulated by specific signals, such as signals from nutrient or stress sensing, metabolism, translation, reproduction, telomeres, etc. [3]. Systems biology can build on these findings and predict the critical players and combinatorial effects. Large-scale approaches are generally used to monitor candidate genes at the same time, often with or without perturbation to the system. 

### 3.1 Genetics-Based Approaches

Two independent genome-wide RNAi screens for longevity genes in *C.elegans* have found 89 and 23 longevity genes respectively, from more than ten thousand clones [28, 29], but there is still a significant false negative ratio [29]. In addition, given that these screens do not identify any gene whose function promotes longevity, the number should probably be at least double. Candidate gene-based or genome-wide association study (GWAS) have identified single nucleotide polymorphism (SNPs) in FOXO1A and FOXO3A contributing to the longevity of human populations [30-33]. A further genetic approach to predicting possible genetic cause of aging uses the identification of expression quantitative trait loci (eQTL), and has identified associations between SNPs and genes with expression changes during aging [18]. 

### 3.2 Expression Profile-Based Approaches

A different, but straightforward, approach to predicting aging regulators uses the profiles of transcriptional or translational changes induced by genetic or environmental perturbations that alter lifespan. Comparison of transcriptional profiles between long-lived *daf-2 *or *age-1* mutants and wild-type or the *daf-16; daf-2* double mutant showed that potential downstream targets of *daf-16* are also aging regulators like *daf-16* itself. Additional comparisons of time course data using *daf-2* RNAi, *daf-2* and *daf-16* double RNAi and wild type reduced the false positive rate in the prediction of aging regulators [34]. A similar experiment that measured translational profile changes by *ifg-1 RNAi*, the *C. elegans* ortholog of eIF4G, found that factors that suppress the longevity induced by *ifg-1* RNAi can be predicted by detecting differentially translated genes [23].

Another common procedure is based on the assumption that the upstream regulators responsible for transcriptional changes during aging potentially regulate aging. Through searching differentially expressed genes, enriched transcription factor (TF) binding motifs enriched on the promoters these genes, NF-kB and elt-3/elt-5/elt-6 GATA transcriptional circuit have been identified as aging regulators in mammals and *C. elegans,* respectively [35, 36]. 

Approaches based on transcriptional profiles are quick to implement, but have certain limitations, for example, the overrepresentation of TF motifs does not necessarily indicate a causal relationship. Even when the predicted factors are necessary for the expression change, they are not necessarily sufficient to cause the change. 

### 3.3 Network-Based Approaches

Based on these large-scale molecular interactions data, such as protein-protein interactions (PPIs), genetic interactions, TF-target interactions, and miRNA-target interactions, molecular networks can be used to visualize the relationships among a gene set, with genes represented as nodes and their molecular interactions as edges. Topological features of a network can often reveal the most critical regulators as hubs, or nodes with the most links, and the functional units/neighborhood among genes as the network modules, within which nodes are densely connected and in between which the nodes are relatively loosely connected. 

Managbanag *et al.* [37] have found that the genes linking the known longevity genes in *Saccharomyces cerevisiae* through PPI shortest paths are more likely to be aging regulators. Utilizing a human PPI network, Bell *et al.* [38] found that genes within a subnetwork consisting of human aging regulators, human homologs of aging regulators identified in invertebrates, and their one-step neighbors are more likely to be hubs and more connected to each other than expected for the rest of the PPI network. Budovsky *et al*. [39] also showed that hubs in the PPI network connecting human homologs of lifespan modifiers were often associated with age-related diseases. Focusing on the modularity of the aging/longevity network, we have shown that the subnetwork of genes with positively or negatively correlated transcriptional changes during aging largely exist in a small number of network modules. In particular, the modules showing negative expression correlation with each other during aging correspond to alternative temporal cellular states, such as proliferation versus differentiation and reductive metabolic versus oxidative metabolic, and the genes connecting modules tend to be enriched for transcriptional regulators and lifespan/aging modifiers [40, 41].

Network analyses additionally revealed systems level relationships between age-related diseases and the aging regulators. Miller *et al.* [42] used a weighted gene co-expression network to identify transcriptional networks in Alzheimer's disease (AD) and found a significant association between gene expression changes during the progression of AD and those during normal aging. Wang *et al.* [43] constructed a human disease-aging network to study the relationships between aging genes and genetic disease genes. This study showed that disease genes located close to aging genes have central positions in the PPI network. 

Network approaches are instrumental in discerning global properties of aging/lifespan regulators, making computational predictions and inferring the modularity and relationships of various aging regulators. However, they should be applied with great caution as to avoid bias introduced by the literature, the lack of spatial and temporal information, or the limited coverage of the network [44]. 

## 4 EPIGENETIC REGULATION OF AGING

In addition to gene expression changes, the states of epigenetic modifications have emerged to be significantly important in modulating lifespan (see the accompanying review by Liu and Zhou in this issue [45]). Epigenetic modifications include DNA and histone modifications that are potentially heritable and reversible without changing the genetic code [46]. With the application of recent high-throughput approaches, such as bisulfite sequencing, ChIP-seq or ChIP-chip, etc. (Section 1), epigenetic controls have become well-recognized as important regulatory mechanisms during the lifetime of an organism [46, 47]. For example, using the anti-O-GlcNAc ChIP-on-chip whole-genome tiling arrays on *C.elegans,* Love *et al*. [48] found 800 genes displaying differential cycling of O-GlcNAc which have functions closely related to aging. By examining DNA methylation at CpG sites throughout the human genome, Hernandez *et al.* [49] identified hundreds of CpG sites with levels of DNA methylation in the human brain highly correlated with chronological age.

Many regulators of histone modifications have been found to be associated with longevity in worms and fruitflies [28, 50, 51]. Recently, several excellent studies further highlight the link between histone modifications and the aging process Fig. (**3**). In particular, Peleg *et al.* [52] found that deregulated acetylation of histone H4 lysine 12 (H4K12) may represent an early biomarker of an impaired genome environment and reduced cognitive ability in the aging mouse brain. Additionally, blocking the loss of H4K12 acetylation incurred by increased histone deacetylase 2 activity in the brain of Alzheimer mouse model could effectively block the onset of the disease [53]. The Sir2 histone deacetylase regulates the replicative lifespan of yeast by acting on histone H4K16 at subtelomeric regions and near the ribosomal DNA [54]. Increasing levels of SIRT1, a mammalian homolog of Sir2, promoted genomic stability and delayed aging-related gene expression changes in mice, in part, by decreasing histone acetylation [55]. The genome-wide binding profiles of the SIRT1, analyzed by ChIP-chip, revealed that genes disassociated with SIRT1 upon DNA damage in mouse embryo stem cells significantly overlap with genes derepressed in the aging mouse brains, suggesting a role of SIRT1 in keeping gene silenced in the young cells [55]. Greer *et al.* have shown that members of the H3K4 trimethylation complex regulated lifespan in a germline-dependent manner in *C. elegans* [56]. We have found that a H3K27 demethylase UTX-1 regulated *C. elegans* lifespan by changing the H3K27me3 states of genes in the insulin/IGF-1 signaling pathway [57]. Together, these works indicate that histone modifications can be equally important as gene expression as both markers and modifiers of aging.

Unlike gene expression profiling, comprehensive profiling of the epigenetic landscape during aging has been hindered by the identification of numerous epigenetic marks and their combinational codes [58-60]. Currently, experimental limitations allow only for the selection of a specific histone or DNA modification of interest, for example, whose modification enzyme is known to affect aging/lifespan, and generate a genome-wide profile for the particular modification. Future high-throughput technologies that can simultaneously probe multiple or all epigenetic modifications will greatly expedite our understanding of the aging epigenome. In the meantime, focus on the epigenetic changes that directly determine gene expression [61] may greatly simplify the task of mapping the aging epigenome.

## 5 FUTURE DIRECTIONS

Systems biology has made great progress during the last decade, mainly as a platform to study complex biological problems. Systems biology has also made great achievements in aging through profiling mRNA, miRNA and protein changes during aging, inferring the relationships among aging-associated genes and predicting unknown aging regulators. However, it is far from enough. 

At least two aspects need to be addressed using a system biology approach in aging research. First, although many different pathways, compartments or processes are known to be closely related to aging, such as the IIS pathway, autophagy, mitochondria, oxidative stress response and so on, it remains unclear as to how they interact, are co-regulated and balanced during aging. To provide a glimpse of this problem, we visualized the network communities among the known aging regulators based on entries in the GenAge database [62, 63] Fig. (**4A**). Utilizing either protein interaction network data Fig. (**4A**) or literature co-citation data Fig. (**4B**) as a network template, the aging regulator network appears to robustly consist of four major parts: 1) signaling pathways sensing nutrients and controlling growth and proliferation (green nodes), DNA damage response for maintaining integrity of the genome (red nodes), mitochondria and oxidative stress response (yellow nodes), and ribosome and translation (blue nodes). It is obvious that the first two are intensively linked and closely entangled, while the latter two are relatively independent processes with only few links connected to the first two processes. Also, it is interesting to note that, by comparing the molecular interaction-based network with the co-citation network, the role of autophagy and protein transport in aging might be either over-estimated due to study bias or under-estimated by the incompleteness of the molecular interactions among these genes. 

Second, although high-throughput data on different layers of the living system Fig. (**2**) can now be easily obtained, it remains obscure as to how information flows or exchanges across these layers to arrive at the alternative “old/aging” state of the molecular network from the young state, what events cause the state transition and what are the network circuitry and epigenetic events locking the network in the aging state.

## Figures and Tables

**Fig. (1):**
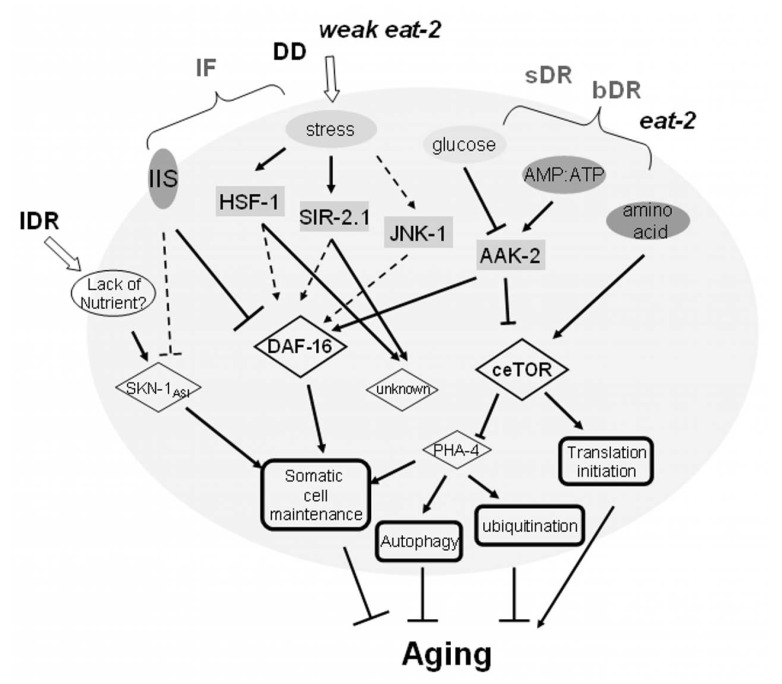
**A brief summary of the effects of dietary restriction (DR) on aging in *C.elegans***. lDR (liquid food DR), IF (intermittent fasting), DD (dietary deprivation), sDR (solid food DR), bDR (bacterial DR) and *eat-2* are different DR strategies or mimics, as indicated in [4, 5]. Dashed lines denote known relationships between the two nodes which have not been demonstrated in DR.

**Fig. (2):**
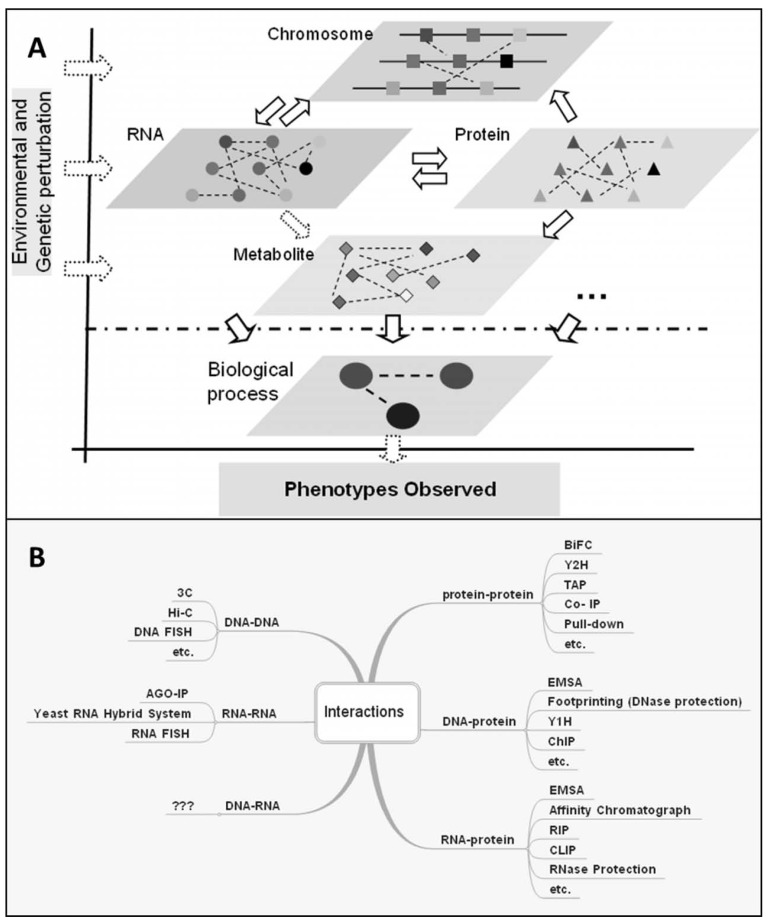
**Different layers of molecular interactions in a biological system**. **A**) Life as a system is composed of information from different layers, modified based on [6]. **B**) Different approaches for mapping interactions within and between different layers. 3C (Chromosome Conformation Capture), Hi-C (High-throughput Chromosome Capture), FISH (Fluorescence In Situ Hybridization), AGO-IP (Argonaute Immunoprecipitation), BiFC (Bimolecular Fluorescence Complementation), Y2H (Yeast Two-Hybrid), TAP (Tandem Affinity Purification), Co-IP (Co- Immunoprecipitation), EMSA (Electrophoretic Mobility Shift Assay), Y1H (Yeast One-Hybrid), ChIP (Chromatin Immunoprecipitation), RIP (RNA Immunoprecipitation), CLIP (Cross Linking and Immunoprecipitation).

**Fig. (3):**
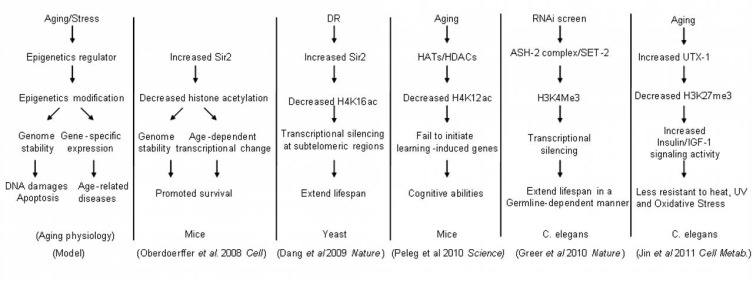
**A summary of data linking epigenome to aging** [52, 54-57].

**Fig. (4):**
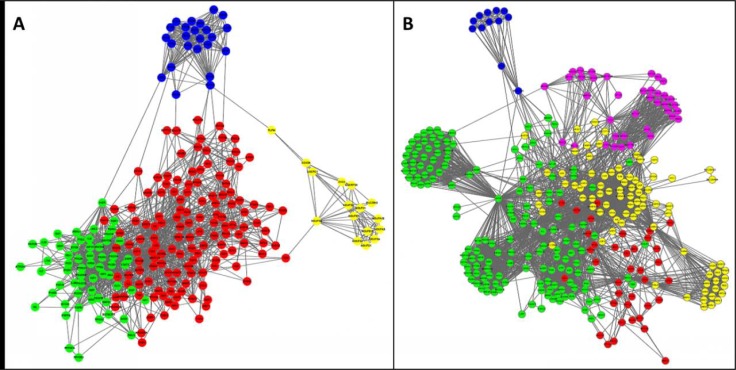
**Network communities among known aging regulators in human and model organisms based on two different interactome datasets**. Nodes include human aging regulators and human homologs of aging regulators in worm, fly and mouse from GenAge [62, 63]. Clusters or communities in the networks were extracted by the MCL algorithm [64] and only top clusters with more than 10 genes for each network are shown, and different clusters with similar functional enrichment are merged. (**A**) The network based on a protein functional interaction network [65]. (**B**) The edges in the network represent cocitation of the two genes together in at least 2 PubMed abstracts under the context of aging, i.e. also co-cited with “aging”, “ageing”, “lifespan”, “life span” as calculated by Cociter (http:// www.picb.ac.cn/ hanlab/cociter). In both graphs, the enriched functions within the gene clusters are coded by the colors of the nodes: green - signaling pathways, red - DNA damage response, yellow - mitochondria function and oxidative stress response, blue - ribosome and translation related genes, and purple - protein localization, transport and autophagy.
